# Self-aligned, full solution process polymer field-effect transistor on flexible substrates

**DOI:** 10.1038/srep15770

**Published:** 2015-10-26

**Authors:** Yan Yan, Long-Biao Huang, Ye Zhou, Su-Ting Han, Li Zhou, Jiaqing Zhuang, Zong-Xiang Xu, V. A. L. Roy

**Affiliations:** 1 Department of Physics and Materials Science, City University of Hong Kong, Kowloon Tong, Hong Kong SAR; 2 State Key Laboratory for Millimeter Waves, City University of Hong Kong, Kowloon Tong, Hong Kong SAR.; 3 Department of Chemistry, South University of Science and Technology of China, Shenzhen 518055, China; 4 Institute for Advanced Study, Shenzhen University, Shenzhen, Guangdong, 508060, P.R. China

## Abstract

Conventional techniques to form selective surface energy regions on rigid inorganic substrates are not suitable for polymer interfaces due to sensitive and soft limitation of intrinsic polymer properties. Therefore, there is a strong demand for finding a novel and compatible method for polymeric surface energy modification. Here, by employing the confined photo-catalytic oxidation method, we successfully demonstrate full polymer filed-effect transistors fabricated through four-step spin-coating process on a flexible polymer substrate. The approach shows negligible etching effect on polymeric film. Even more, the insulating property of polymeric dielectric is not affected by the method, which is vital for polymer electronics. Finally, the self-aligned full polymer field-effect transistors on the flexible polymeric substrate are fabricated, showing good electrical properties and mechanical flexibility under bending tests.

As the rapid improvement in the performance of polymer field-effect transistors (PFETs), the manufacturing of PFETs based on low-cost and large-area solution-processing techniques on the flexible substrate is being considered, which can provide new opportunities for potential applications in organic electronics such as flexible active-matrix displays and radio-frequency (RF) identification tags (RFID)^1^,^2^,^3^,^4^,^5^,^6^,^7^. Therefore, the research for the full polymer FETs on the plastic substrates which avoid using vacuum deposition for small molecules and metal electrodes are essential^8^,^9^,^10^,^11^,^12^,^13^,^14^,^15^. In order to realize the full polymer FETs, the traditional metal electrodes made up of Au, Ag or Al should firstly be replaced by conducting polymers. However, it is challenging to control the flow of conducting polymer inks on the surfaces for the patterned polymer electrodes fabrication. There are reports on low-cost fabrication of solution-processed PFETs using different patterning techniques such as photolithographic patterning, soft lithographic stamping, micromolding in capillaries, screen printing, and inkjet printing^14^,^16^,^17^,^18^,^19^,^20^. Meanwhile, patterning the organic active layer is one of the useful methods to avoid crosstalk, and can be achieved by oxygen plasma etch, photolithography or contact printing^21^,^22^,^23^,^24^. Nevertheless, the conditions become more complicated due to materials limitations when the device is on the flexible polymer substrates. High temperature and etching effects should be avoided during the patterning process that would damage the plastic film structure and property. In addition, for the process of electrode patterning on the dielectric surface, the insulating property of polymer dielectric should not be affected, which is vital for the device performance. For instance, among various patterning techniques, the most commonly used approach for overcoming limitations of resolution is to modify substrates with a predefined selective wettability pattern. On this regard, the widely used conventional technique for the fabrication of selective surface energy region is the photolithography patterning, followed by etching treatment such as reactive ion etching or wet chemical etching^25^,^26^. Unfortunately, these techniques are not suitable for polymer structures due to the plasma-induced degradation and the lack of effective anisotropic etching. Therefore, there techniques are suitable for rigid substrates such as silicon or glass. For the full polymer FETs with top gate architecture, the etching process brings serious destruction on the polymeric insulator layer and has an adverse impact on the overall device performance. Patterning two types of self-assembly monolayers (SAMs) on the substrate with different wettability have also been utilized to fabricate FETs with patterned active layer^27^,^28^. However, reaction sites on the substrates are necessary to react with the SAMs for realizing the surface modification through molecular functional groups to form a tight connection^29^,^30^,^31^,^32^,^33^. Unfortunately, the reaction sites (usually hydroxyl group) on the plastic substrates or polymer insulators are not sufficient or even do not exist compared with inorganic surfaces such as SiO_2_ or Al_2_O_3_. Without such reaction sites, SAMs modification of polymer becomes almost impossible. Therefore, using an appropriate patterning technique for full polymer FETs on flexible plastic substrates with the following properties are necessary for technological applications: i) Low-cost and suitable for large-area and solution-processing. ii) Good compatibility with low-temperature processing to protect the plastic flexible substrate and the underlying polymer multilayer structures. iii) A lesser amount of etching effect to prove flattened interface quality for plastic substrates and polymeric insulator layer. In the device fabrication process, the etching effect raises interface roughness of substrates as well as reduces the thickness of plastic substrate and dielectric layer which degrades the device performance.

Here, we demonstrate full polymer FETs fabricated through four-step spin-coating process on the flexible substrate by employing the confined photo-catalytic oxidation (CPO) method to pattern the source/drain (S/D) electrodes on poly(ethylene terephthalate) (PET) substrates and gate electrodes on poly(methyl methacrylate) (PMMA) polymeric films, respectively^34^. A patterned hydrophobic/hydrophilic region is formed on the PET substrates and PMMA dielectric layer surface by UV light motivated oxidation reaction through the patterned glass masks. With the forming of selective wettability patterned regions on flexible PET surfaces, the polymer S/D electrode array based on poly(3,4-ethylenedioxythiophene) polystyrene sulfonate (PEDOT/PSS) solution was deposited on the treated film by spin-coating and dipping. The contact angle of polymer substrates reduced from 73° to 15° for PET and from 85° to 19° for PMMA, respectively. The active layer poly(3-hexylthiophene-2,5-diyl) (P3HT), dielectric layer PMMA and final PEDOT/PSS gate electrode are fabricated by spin-coating and dipping method. The surface morphology of the treated polymeric surfaces was measured by atomic force microscope (AFM), and a negligible etching effect was observed. Additionally, the attenuated total reflectance Fourier transform infrared (ATR-FTIR) and X-ray photoelectron spectroscopy (XPS) results also reflect that the molecular sulfate salt group layer has been implanted covalently onto the surface of polymeric surfaces that reduces the etching effect for polymeric films. In addition, the CPO method is low cost and suitable for large-area and low temperature solution-processing. In this work, all the materials including PET, P3HT, PMMA and PEDOT/PSS are low-cost polymers and all the fabrication procedures are solution-processed at low temperature. The self-aligned full polymer FETs on the flexible PET substrate show good electrical properties and flexible stability under bending tests.

## Results

### Full polymer FETs fabrication

**Figure 1:**
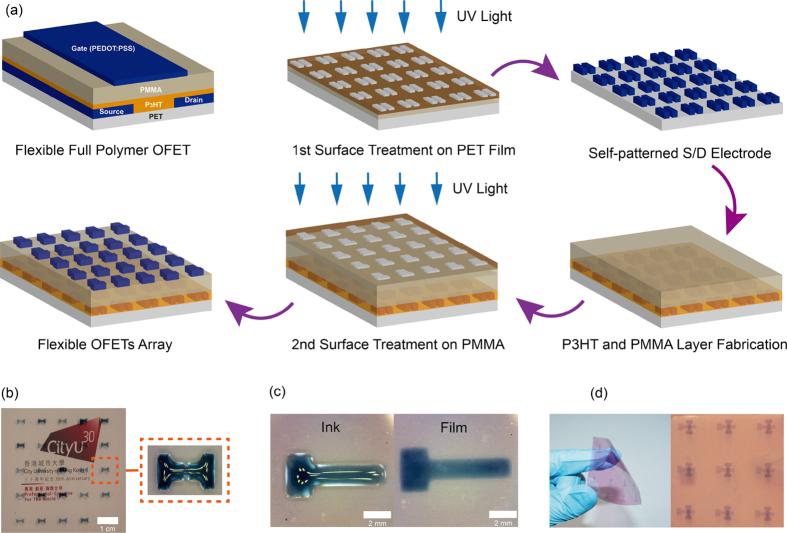
Device fabrication process (a) Schematic diagram depicting a polymer FET device and the fabrication process of bottom-contact top-gate full polymer FETs on flexible PET. (**b**) An optical image of the PEDOT/PSS solution self-aligned on the patterned S/D regions by the dipping method and (**c**) PEDOT/PSS (1:1) solution self-aligned on the patterned gate regions by the dipping method and (**d**) the flexible polymer FETs based on PET substrates.

The fabrication process of bottom-contact top-gate full polymer FETs on flexible PET substrates is described in Fig. 1a. The persulfate salt aqueous solution with amount (30 wt%, 500 μl) was dropped on the PET surface using a micro-syringe, and covered by a patterned mask made of quartz glass. The solution spread to form a thin liquid layer (around 2 μm) due to the pressure from patterned mask. The sandwich unit was irradiated by UV light from the top at room temperature for 10 mins. After the irradiation, patterned hydrophobic and hydrophilic regions were formed on the treated PET and a self-pattern polymer electrode array was formed. Then, the polymer S/D electrodes based on PEDOT/PSS film were formed on the treated film by either spin-coating or dipping process. Due to the different wettability of the treated PET surface, the PEDOT/PSS solution would only exist on the hydrophilic areas to form the S/D electrode array. An optical image of the PEDOT/PSS solution self-aligned on the patterned S/D regions by the dipping method is shown in Fig. 1b. The semiconductor polymeric P3HT was subsequently spin coated on the patterned PET film, followed by spin-coating the PMMA dielectric layer on the top. After then, the same surface treatment and polymer electrode fabrication process were repeated on the polymer insulator surface to form the polymer gate electrode array. An optical image of the PEDOT/PSS solution self-aligned on the patterned gate regions by the dipping method and finally flexible PFETs based on PET substrates are shown in Fig. 1c,d.

### Polymeric surface modification and characterization

**Figure 2:**
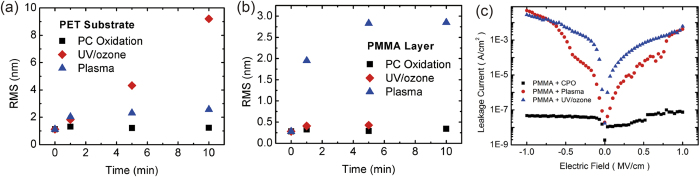
Characterization of polymeric film. (**a**) RMS roughness dependence on the reaction time for confined photo-catalytic oxidation method, UV/ozone, and O_2_ plasma treatment on PET and (**b**) PMMA substrates. (**c**) Leakage current as a function of the applied electric field of PMMA insulator treated with CPO (10 mins), Plasma (10 mins) and UV/ozone (8 mins).

**Figure 3:**
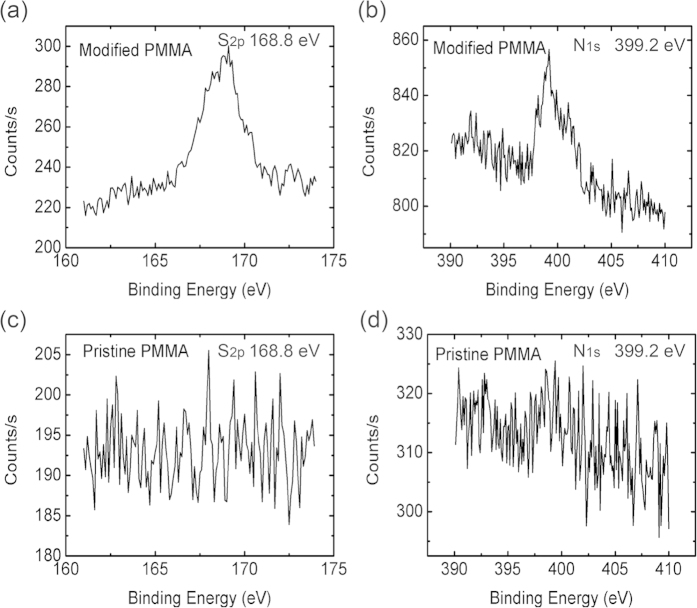
Characterization of polymeric film. XPS spectra of modified and pristine PMMA surfaces: S_2p_ peak (**a**,**c**); N_1s_ peak (**b**,**d**), the upper: after modified; the lower: before modified. ((NH_4_)_2_S_2_O_8_, 30 wt%; irradiation time: 10 mins).

The hydrophilicity of polymer surface is determined by chemical composition and related surface topographical structure. As we know, UV/ozone and plasma treatment are very common approaches not only to modify the surfaces to induce the hydrophilicity of both organic and inorganic materials but also to clean a variety of contaminants on the surface^35^,^36^,^37^,^38^,^39^. Here we studied the PET and PMMA surface morphology and related etching effect as a function of surface treatment reaction time by O_2_ plasma treatment, UV/ozone treatment, and confined photo-catalytic oxidation method. The exposure time is decided by the change in contact angle measurements. Considering the contact angle change is not an instantaneous decrease after the surface treatment with CPO, O_2_ plasma and UV/ozone, we measure the gradual change in the contact angle with time. An exposure of 10 mins is enough for obvious surface energy changes. Therefore, we record and compare the RMS and leakage current change at 0, 1, 5, and 10 mins. The AFM height image of PET and PMMA film morphology changes with reaction time for the three treatment methods are depicted in Supplementary Figure 1. Figure 2a,b show the variation in root-mean-square (RMS) roughness with changes of reaction time, demonstrating that conventional O_2_ plasma treatment and UV/ozone treatment create an obvious etching effect on polymer surface and increase the treated surface roughness. Especially, for the sample of 10 mins UV/ozone treatment on PET surface, the RMS increases to 10 nm and this is an obvious drawback for forming a smooth polymer layer on the top. Moreover, for the sample of 10 mins UV/ozone treatment on PMMA surface, no data is shown here since the 300 nm thick PMMA dielectric layer coated on the silicon substrate was totally removed due to the strong etching effect. However, no surface topological changes were observed in confined photo-catalytic oxidation method. In Supplementary Figure 2, the AFM images of PEDOT/PSS film on treated PET with confined photo-catalytic oxidation method for 10 mins shows a smooth conductive polymer film with RMS = 1.12 nm, which is similar to low roughness pristine PET surface. At the same time, from the gravimetric analysis, no obvious changes in smoothing effect were observed in confined photo-catalytic oxidation treated samples (by electrobalance with accuracy of 0.00001 g). These results demonstrate that the surface etching or smoothing effect could be excluded during the CPO process. Notably, for the thin functional insulator layer (200 ~ 300 nm), the UV-ozone or plasma treatment is destructive for the film’s electrical property. Figure 2c shows the leakage current as a function of the applied electric field of 300 nm thick PMMA insulator treated with CPO, plasma and UV/ozone. The insulating property of PMMA is significantly affected with the plasma and UV/ozone treatment, in the end adversely influencing the field-effect performance. This shows that CPO based surface treatment is more suitable for thin polymeric insulator surface, which ensures the feasibility of electrode pattern on the top. In the PFETs fabrication process, a reduced amount of etching effect proves a flattened interface quality therefore avoids the damage to plastic substrates and polymer insulator layer, which is critical for device performance. Surface chemical structure with the thickness around 1–10 mm could by characterized by ATR-FTIR and the interface chemical element changes for relatively thinner thickness (within the order of 1–5 nm) could be investigated by XPS. Here we investigate PMMA samples with and without CPO treatment to analyse the oxidation depth using ATR-FTIR and XPS techniques. As shown in Fig. 3a,d, two new peaks of S and N elements are observed in the XPS spectra of PMMA samples with material persulfate ammonium ((NH_4_)_2_S_2_O_8_) as reaction oxidant in Fig. 3a,b. The peaks at 168.8 eV (S_2p_) and 399.2 eV (N_1s_) in treated PMMA samples are respectively attributed to the S (SO_4_^−^) and N (NH_4_^+^). However, the two peaks are not observed in the XPS spectra of pristine PMMA samples in Fig. 3c,d. The above fact implies that sulfate anion groups from the reaction solution are implanted onto the outmost surface which increased the surface hydrophilicity effectively^34^. Additionally, for the ATR-FTIR measurement, no change is observed in treated and untreated PMMA samples as shown in Supplementary Figure 3. The different results from XPS and ATR-FTIR reveal that the molecular sulfate salt group layer is implanted covalently onto the outmost surface of PMMA samples. Elements could not be detected by ATR-FTIR as the sulfate salt layer is quite thin, which is also consistent with the gravimetric measurement. The CPO method is suitable for a wide range of common available polymeric materials. Regarding substrates, we have tested three kinds of substrates such as polyethylene terephthalate (PET), polyethylene (PE) and polyethylene naphthalate (PEN) and an obvious change in contact angle has been observed. Regarding gate dielectrics, commonly used polymeric dielectric films such as polystyrene (PS), poly-4-vinylphenol (PVP), and poly (methyl methacrylate) (PMMA) have also been successfully modified with CPO method. Contact angles of pristine and CPO treated PS, PVP, PMMA, PET, PE, and PEN samples are given in Supplementary Figure 4.

### Various channel length

**Figure 4:**
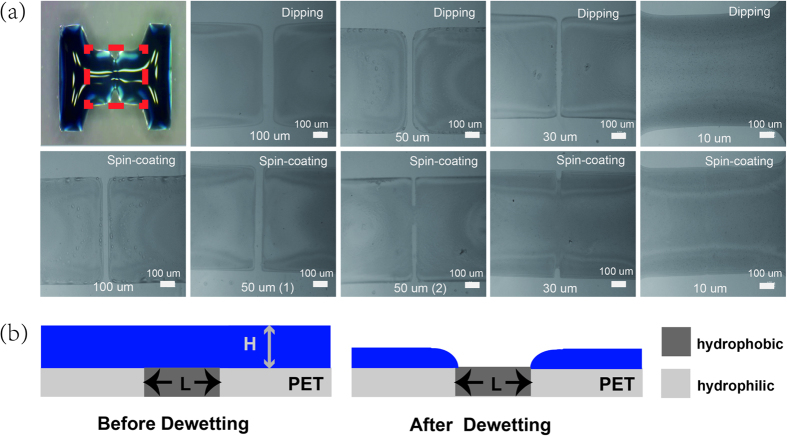
Various channel length. (**a**) Photographs of various PEDOT/PSS (1:1) S/D electrodes patterning by dipping and spin-coating method. (**b**) Schematic diagram depicting PEDOT/PSS solution splitting on patterned hydrophobic/hydrophilic region.

Figure 4a shows the optical images of channel regions with various channel length. For the channel length less than 30 μm, the solution cannot split to form a clear channel for both the dipping and spin-coating method. However, compared with dipping and spin-coating method, the dipping method shows better uniformity in short channel conditions. For instance, the samples with channel length of 50 μm, the patterned region is easy to collapse with spin-coating method and the trend becomes more obvious when the channel length is 30 μm as shown in Fig. 4a. Several factors such as solution concentration, viscosity and volume are vital to achieve separation of PEDOT/PSS solution. A simple model is employed here to explain the fluid-dynamical process^25^. The model should satisfy following two conditions: The liquid volume is assumed constant and the gravity is neglected here. The model suggests that the separation occurs when the film thickness is less than the critical thickness *H*_critical_. In our experiment, the water in the PEDOT/PSS solution evaporates to enhance the concentration and viscosity of the conductive ink. As shown in Fig. 4b, the droplet shows complete separation on the hydrophobic surface when the film reaches its critical thickness *H*_critical_ for dewetting before the viscosity exceeds a critical value *η*_critical_. Therefore, dewetting occurs more easily by decreasing the ink concentration and reducing the liquid volume. The concentrated ink and more solution volume lead to the condition of critical viscosity. However, the low concentration or less volume ink form a much thinner film during the drying process before it comes to *η*_critical_. Additionally, the contact resistance of the device is influenced by the conductivity of PEDOT/PSS layer. A high concentration and large volume PEDOT/PSS solution attribute to lower contact resistant and better electrical properties. Therefore, it is a compromise for the choice of conductive polymer volume and concentration. To obtain a device with short channel (submicrometre) and low contact resistance, it is not only essential to control the liquid viscosity, concentration, surface-energy difference and the drop volume, but also the surface-energy barrier^25^.

### Electrical Performance

**Figure 5:**
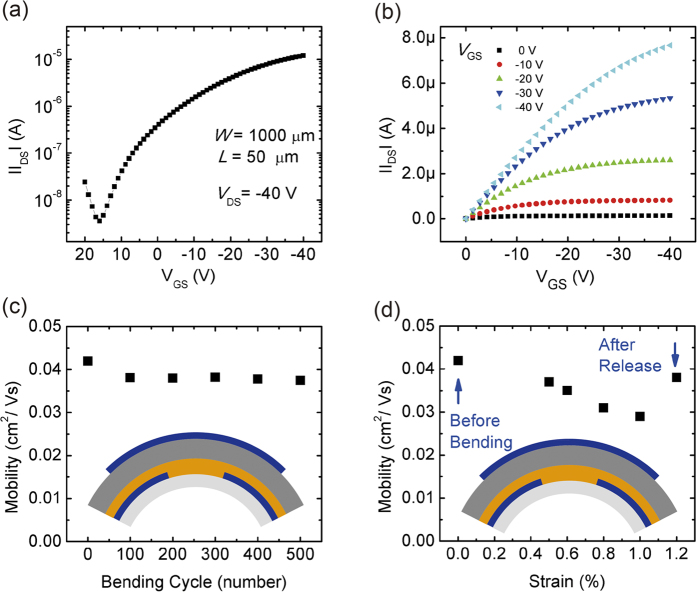
Characteristics of FETs. (**a**) Transfer curve (V_DS_ = −40 V) and (**b**) Output curve of flexible full polymer FETs (W = 1000 μm, L = 50 μm, electrode fabricated by dipping method). Flexible stability characteristics of (**c**) Mobility as a function of bending cycles (strain = 1%) and (**d**) Mobility as a function of tensile strain.

Figure 5a,b show the electrical properties of full polymer FETs (*W/L* = 1000 μm/50 μm) on the flexible PET substrate. Electrodes were fabricated by dipping method with PEDOT/PSS (1:1) solution. The device displays typical p-type FET performance, exhibiting a threshold voltage (*V*_*Th*_) of −1 V, an *I*_*on*_*/I*_*off*_ ratio of about 3.5 × 10^3^, a saturation mobility of around 0.042 cm^2^/Vs, showing a similar performance of P3HT based top-contact and bottom-gate FET with 100 nm SiO_2_ as insulator layer and Au as electrode in Supplementary Figure 5 and the bottom-contact top-gate device with Au as electrode, P3HT as active layer and PMMA as dielectric layer (without the CPO treatment) in Supplementary Figure 6. As shown in Fig. 4a, electrodes fabricated by spin-coating method is more suitable for the channel length large than 50 μm to achieve the uniformity of different devices on the same substrate and the respective transfer curve of FETs (*W*/*L* = 1000 μm/100 μm) is shown in the Supplementary Figure 7. The technique of spin-coating is commonly used and known for reproducible film quality and uniformity. Also, the films thickness can be accurately tuned by regulating spin time and speed. Experiments of sample to sample dispersion of S/D yield, channel length variation and I-V characteristics changes have been performed and the results are shown in the Supplementary Figure 8 and Figure 9. In our work, by employing the confined photo-catalytic oxidation to pattern the polymer electrode, the full polymer FETs fabricated through four-step spin-coating process on the flexible substrate are realized. In addition, we carried out the bending stability test of the flexible FETs devices as illustrated in Fig. 5c,d. The strain is approximated from the values of D = 2R, where D is the PET substrate thickness, R is the bending radius^40^. At first, the flexible devices were bent with a 1% strain repeatedly as shown in Fig. 5c, the flexible device showed excellent mechanical stability during the bending test. After 500 cycles, the performance of the devices did not change significantly. In addition, the flexible transistor was measured at various mechanical bending strains as shown in Fig. 5d. The flexible transistor exhibited slight change in mobility when the mechanical bending reaches 0.5% strain. Moreover, when the devices were measured after release, the performance could be close to the initial state.

## Discussion

We realized full polymer FETs on flexible PET substrates by utilizing the confined photo-catalytic oxidation method to pattern the S/D and gate electrodes on PET and PMMA films respectively. The pattern technique is low-cost, short time, simple operation and suitable for large-area solution-processing techniques with less etching effect and good compatibility for low-temperature processing. In addition, the insulating property of polymeric dielectric is also not affected. The CPO method can be applied for more complicated circuits to form different patterns without destroying the bulk material properties. The flexible full polymer FETs showed good electrical properties and excellent flexibility during the bending test.

## Methods

### Materials

Commercially available polyethylene terephthalate (PET) with a thickness of 200 μm was used as the flexible substrate. Ammonium persulfate (APS), Poly(3,4-ethylenedioxythiophene)-poly(styrenesulfonate) (PEDOT/PSS), poly (methyl methacrylate) (PMMA. Mw = 120000 g/mol) and P3HT (average Mn 54,000–75,000) were purchased from Aldrich. All of the above chemicals and solvents were used without further purification.

### Device Fabrication

Bottom-contact/top-gate all polymer FETs were fabricated on PET substrates. Films were cleaned with de-ionized water firstly. Then, the persulfate salt aqueous solution (30 wt%, 500 μl) was dropped on the PET surface using a micro-syringe, and followed covering with a patterned mask made of quartz glass (*W/L* = 1000 μm/100 μm, 1000 μm/50 μm, 1000 μm/30 μm, 1000 μm/10 μm). The solution was spread to form a very thin liquid layer (around 2 μm) due to the pressure from patterned mask. The sandwich structure was irradiated under UV light (ML-3500S/F, Spectroline) from the topside at room temperature for 10 mins. After the irradiation, the treated PET substrates were cleaned using deionized water for 10 mins and the patterned hydrophobic and hydrophilic regions formed made the self-pattern polymer electrode array possible. Then, the polymer S/D electrode array based on PEDOT/PSS (1:1) solution was formed on the treated film by two methods (spin-coating and dipping) respectively. For spin-coating, the PEDOT/PSS (1:1) solution, spun at 800 rpm for 40 s. Due to the different wettability of treated PET surface, the PEDOT/PSS solution will existed on the hydrophilic areas and form the S/D electrode array. Then, the patterned substrates annealed at 100 °C for 30 mins on a hot plate. The semiconductor polymer P3HT dissolved in xylenes (6 g L^–1^), spun at 1000 rpm for 1 min, followed by an annealing process at 120 °C for 30 mins. A 20 nm thick semiconductor polymeric P3HT was subsequently spin coated on the patterned PET film, followed by spin-coating a 300 nm thick PMMA dielectric layer on the top. PMMA was dissolved in a 50 gL^–1^ n-butyl acetate solution in N_2_ atmosphere, and annealed at 100 °C for 30 mins to remove solvent. After then, a same surface treatment and polymer electrode fabrication process were repeated on the polymer insulator surface to form the polymer gate electrode array, followed by a post annealing step at 100 °C for 60 mins. In our experiments, PET substrates are fixed on a metal plate during the annealing process. In addition, the process was carried out in the nitrogen glove box or under vacuum to avoid the influence of water and O_2_.

### Characterization

Commercially available polyethylene terephthalate (PET) with a thickness of 200 μm was used as the PET samples. PMMA samples were fabricated on the high doped silicon substrate, dissolved in 50 gL^–1^ n-butyl acetate solution, spun at 2000 rpm for 1 min. The samples were modified with the confined photo-catalytic oxidation method using ammonium persulfate (APS) salt aqueous solution (30 wt%) on the surface under UV irradiation for 10 mins through a transparent quartz glass. For the AFM measurement, the samples were treated by O_2_ plasma treatment with Harrick PDC-32G Plasma Cleaner and UVO treatment with UVO (Jelight Inc. UVO-CLEANER) for various time (0, 1 min, 5 mins, 10 mins). The surface morphologies of PET and PMMA surface were measured in air using an AFM (VEECO Multimode V) operating at the tapping mode. The contact angle measurement used A Rame-hart Model 250-F1 Standard Goniometer with DROP image Advanced 2.1. XPS was measured in the ultrahigh vacuum environment using Physical Electronics PHI 5802 with a monochromatic Al Kα X-ray source. ATR-FTIR was measured using VERTEX 70, Bruker. All electrical characteristics of devices were performed at room temperature in the MBraun nitrogen glove box using a Keithley 2612 source meter, Agilent 4155C semiconductor parameter analyzer and HP 4284A LCR meter.

## Additional Information

**How to cite this article**: Yan, Y. *et al.* Self-aligned, full solution process polymer field-effect transistor on flexible substrates. *Sci. Rep.* **5**, 15770; doi: 10.1038/srep15770 (2015).

## Supplementary Material

Supplementary Information
