# Supplementary material for: Self-aligned, full solution process polymer field-effect transistor on flexible substrates
Source: Sci Rep. 2015 Oct 26;5:15770. doi: 10.1038/srep15770 (PMC4620563; doi:10.1038/srep15770)
Supplement: Supplementary Information [file srep15770-s1.doc]

Self-aligned, full solution process polymer field-effect transistor on flexible substrates

Yan Yan, Long-Biao Huang, Ye Zhou, Su-Ting Han, Li Zhou, Jiaqing Zhuang, Zong-Xiang Xu and V. A. L. Roy*


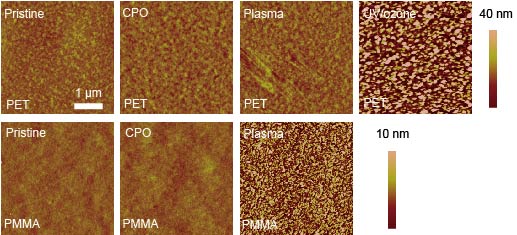


**Supplementary Figure 1:** AFM images of PET and PMMA films. The substrates were treated with confined photo-catalytic oxidation method, UV/ozone, and O_2_ plasma for 10 min.


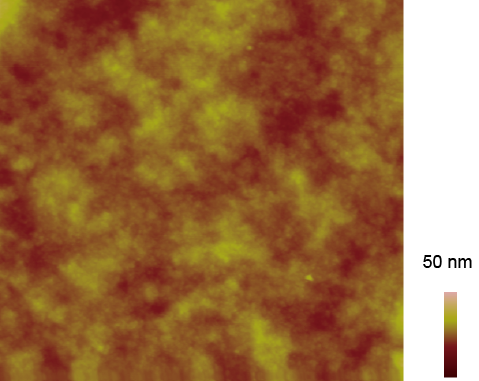


**Supplementary Figure 2:** AFM images of PEDOT/PSS film on treated PET with confined photo-catalytic oxidation method for 10 min.


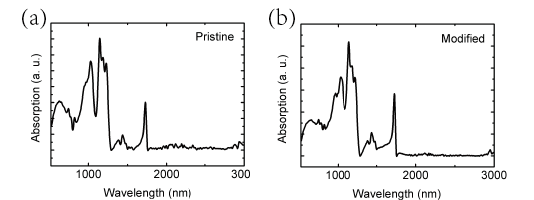


**Supplementary Figure 3:** ATR-FTIR spectra of pristine PMMA and PMMA films modified with confined photo-catalytic oxidation method.


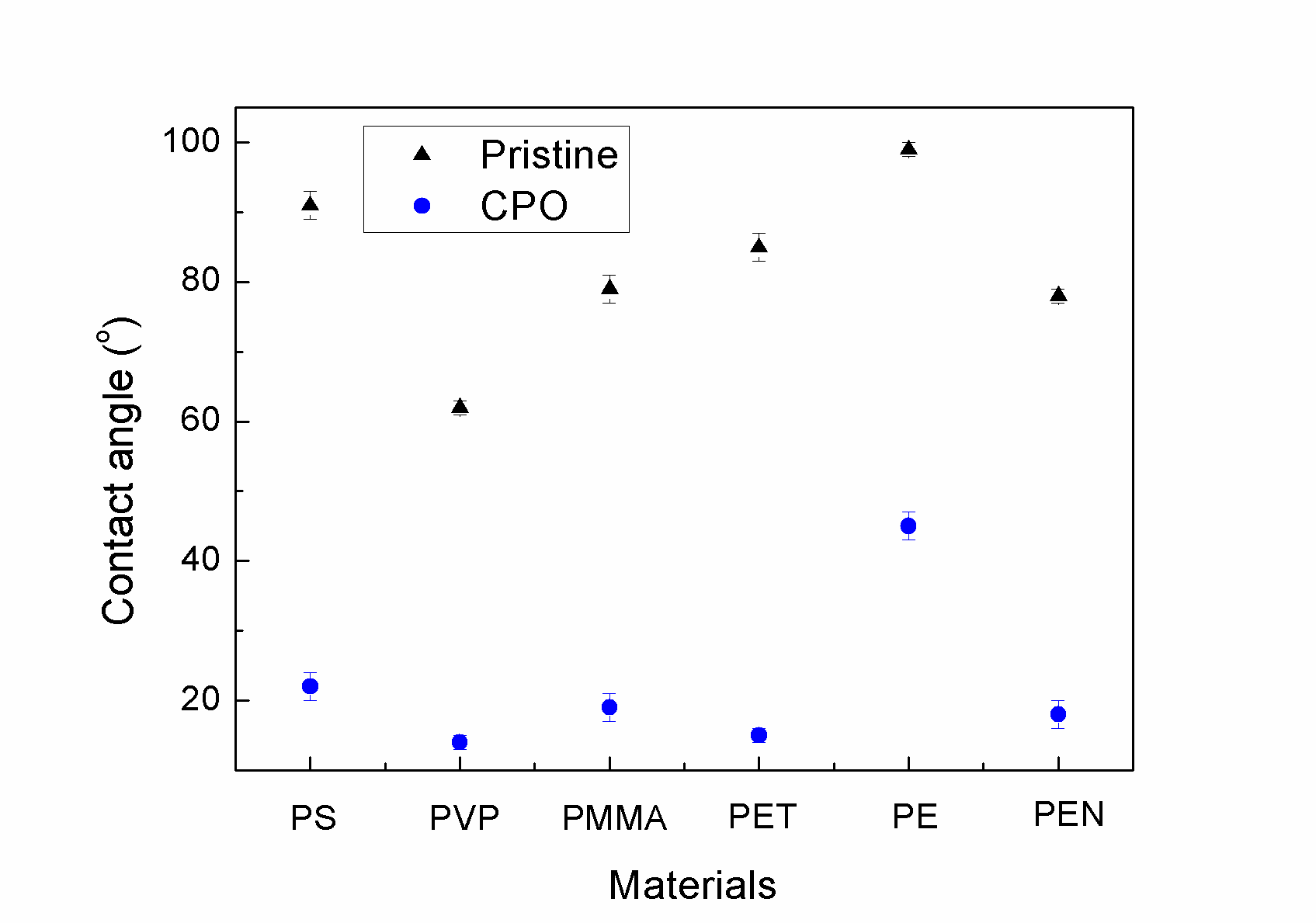


**Supplementary Figure 4:**. Contact angles of pristine and CPO treated PS, PVP, PMMA, PET, PE, and PEN samples


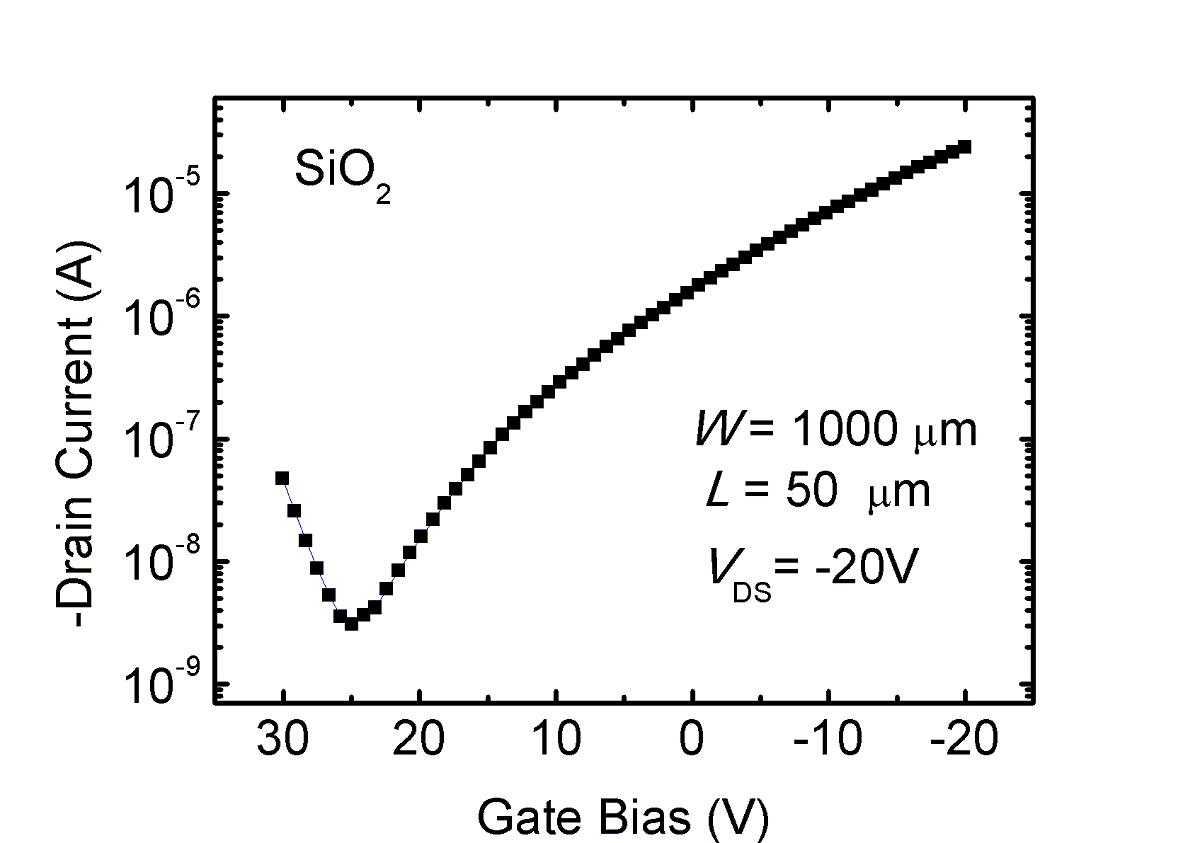


**Supplementary Figure 5:** Transfer curve of P3HT based top-contact and bottom-gate OFET. The thickness of SiO_2_ is 100 nm.


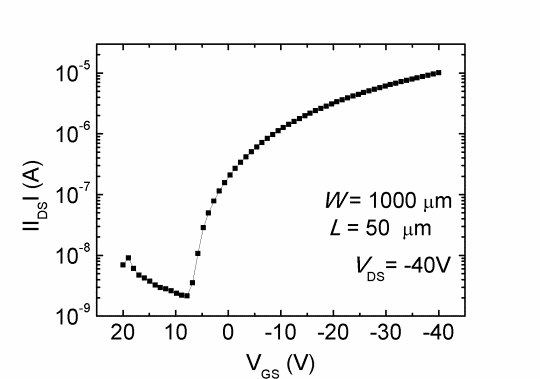


**Supplementary Figure 6:** Transfer curves of bottom-contact top-gate device with Au as electrode, P3HT as active layer and PMMA as dielectric layer (without the CPO treatment).

The device exhibited a threshold voltage (*V_Th_*) of 3.5 V, an *I_on_/I_off_* ratio of about 4.6 × 10^3^, and a saturation mobility of around 0.051 cm^2^/Vs, showing a similar performance of P3HT based bottom-contact and top-gate OFET with the CPO treatment.


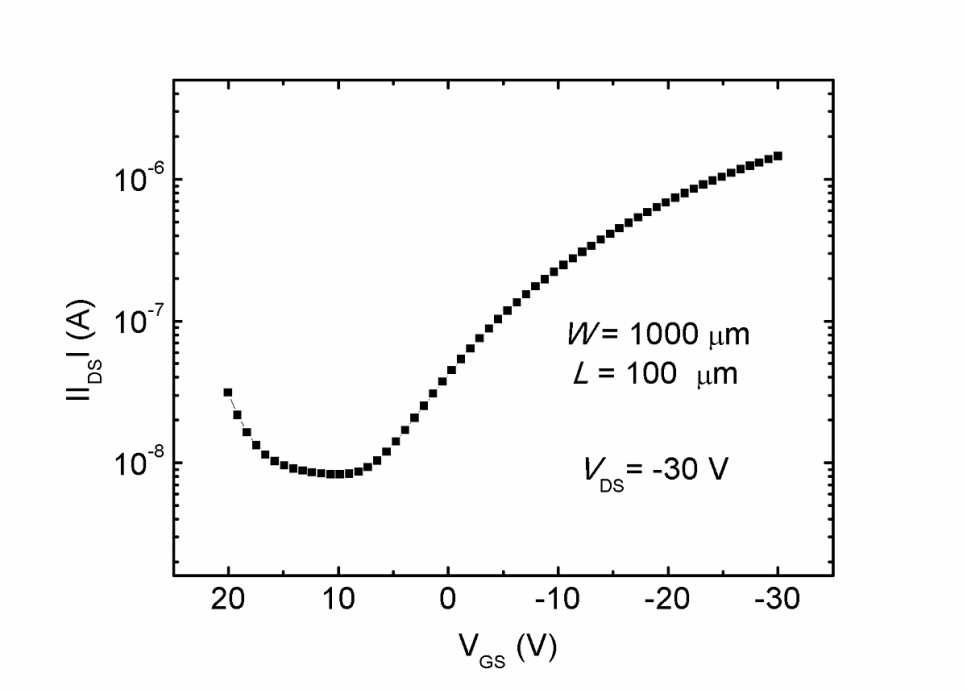


**Supplementary Figure 7:** Transfer curve of flexible full polymer OFETs. Electrode was fabricated by spin-coating method) (W = 1000 μm, L = 100 μm) and The V_DS_ is - 30 V.


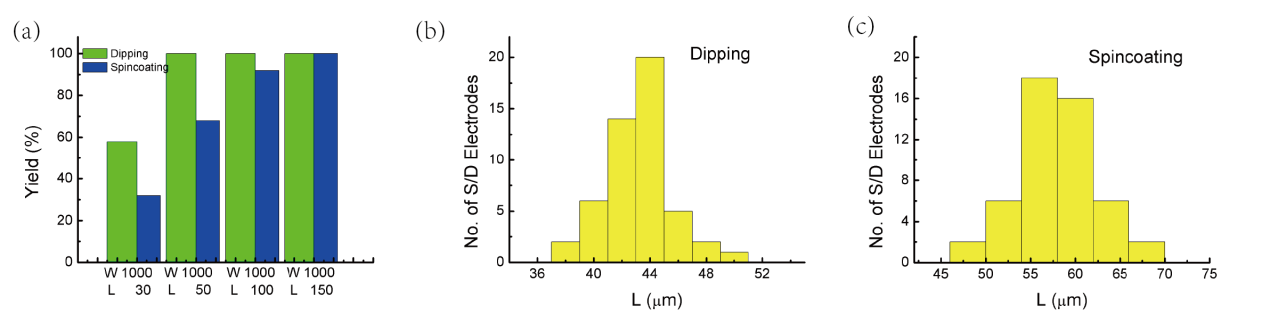


**Supplementary Figure 8:** S/D electrodes yield for electrode fabrication using dipping and spin-coating methods with various channel lengths (L) and channel widths (W).

Supplementary Figure 8a shows S/D electrodes yield for electrode fabrication using dipping and spin-coating methods with various channel lengths (L) and channel widths (W). S/D electrodes of OTFTs with *W* of 1000 μm and *L* of 30, 50, 100 and 150 μm were fabricated utilizing the optimized PEDOT:PSS solution. A total of 50 devices are measured to get the yield. For the dipping method, it displays 100% yield when the L comes to 50 μm, showing a better yield than the spin-coating method (150 μm). Supplementary Figure 8b and 8c displays the channel length variability for S/D electrodes with L of 50 μm. A mean channel length of 44 μm and 58 μm are observed for dipping and spin-coating method respectively. The sample to sample dispersion can be effectively decreased by accurately controlling ink viscosity and volume.


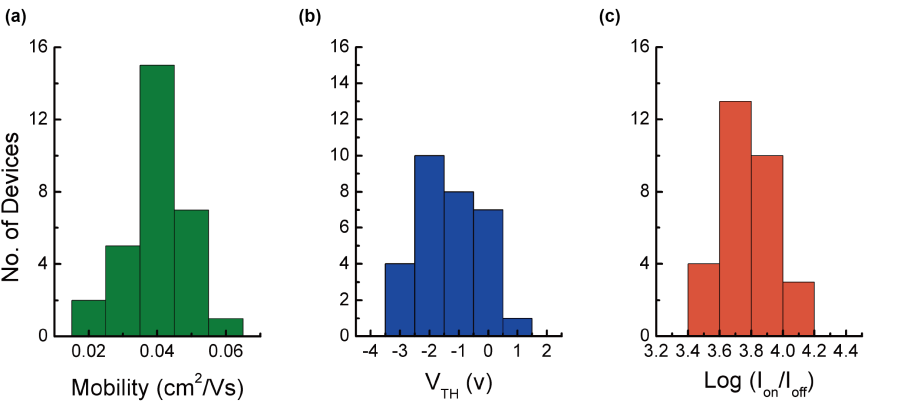


**Supplementary Figure 9:** Distribution of saturation mobility, on/off ratio, and threshold voltage for 30 devices with electrodes fabricated with dipping method.

Supplementary Figure 9a-9c shows the distribution of saturation mobility, on/off ratio, and threshold voltage for 30 devices with electrodes fabricated with dipping method. Devices display typical p-type FET performance, exhibiting a mean threshold voltage (*V_Th_*) of -1.3 V, an *I_on_/I_off_* ratio of over 10^3^, a saturation mobility of around 0.042 cm^2^/Vs. The data has been added in the supporting information.
